# Splenic and portal vein thrombosis following laparoscopic splenectomy in a pediatric patient with chronic myeloid leukemia

**DOI:** 10.1590/S1516-31802006000500008

**Published:** 2006-09-07

**Authors:** Henrique Manoel Lederman, Evan Fieldston

**Keywords:** Splenectomy, Chronic myeloid leukemia, Magnetic resonance spectroscopy, Myeloid leukemia, Esplenectomia, Leucemia miéloide crônica, Ressonância magnética, Leucemia mielóide

## Abstract

**CONTEXT:**

Splenic or portal vein thrombosis is a rare complication following splenectomy.

**CASE REPORT:**

We report a case of splenic and portal venous thrombosis in a 10-year-old girl with chronic myeloid leukemia who underwent laparoscopic splenectomy prior to bone marrow transplant. Clinical suspicion of such thrombosis should be high for patients who have had splenectomy. The diagnosis is confirmed by Doppler ultrasound or contrast-enhanced computed tomography; magnetic resonance imaging magnetic resonance angiography or arteriography can also be used. Proposals for postoperative screening protocols are discussed. Patients with primary myeloproliferative disorders are at increased risk of portal vein thrombosis, independent of surgical intervention, perhaps due to platelet dysfunction resulting from abnormalities of pluripotent stem cells. Marked splenomegaly (with larger draining veins) is thought to increase the risk of thrombosis.

## RESUMO

**CONTEXTO:**

Trombose em veia portal ou esplênica é uma complicação rara pós-esplenectomia.

**RELATO DE CASO:**

Descrevemos um caso de trombose venosa portal e esplênica em uma menina de 10 anos de idade com leucemia mielóide crônica, a qual foi submetida a uma esplenectomia laparoscópica antes de um transplante de medula óssea. A suspeita clínica de tal trombose deve ser alta em pacientes que sofreram esplenectomia, e o diagnóstico é confirmado pelo ultra-som Doppler. Tomografia com contraste, ressonância magnética, angioressonância ou arteriografia podem também ser utilizados. Proposta de protocolo pós-operatório para avaliação de trombose é discutida.

## INTRODUCTION

Splenic or portal vein thrombosis is a rare complication following splenectomy.^1^

We report a case of splenic venous thrombosis in a 10-year-old girl with chronic myeloid leukemia who underwent laparoscopic splenectomy prior to a bone marrow transplant.

Clinical suspicion of such thrombosis should be high for patients who have had splenectomy. The diagnosis is confirmed by Doppler ultrasound or contrast-enhanced tomography; magnetic resonance imaging magnetic resonance angiography or arteriography can also be used.

Proposals for postoperative screening protocols are discussed.

## CASE REPORT

FB, a 10-year-old Caucasian girl who was in her usual state of good health, presented in August 2000 with a distended abdomen and palpable abdominal mass that was found to be a markedly enlarged spleen. Further work-up resulted in a diagnosis of chronic myeloid leukemia. She was treated with hydroxyurea and was scheduled to receive a bone marrow transplant. In preparation for this procedure, laparoscopic splenectomy was performed. Her preoperative complete blood cell test gave a white blood cell count of 18,700/μl, hemoglobin 11.4 g/dl and platelet count of 528,000/μl. The operation was conducted without complications and the postoperative period was marked by left shoulder pain.

On the second postoperative day, the patient's white blood cell count was 55,000/μl and platelet count was 1,144,000/μl. It was felt this increase was consistent with her splenectomy and her dose of hydroxyurea was increased to 750 mg (twice a day). On the fifth postoperative day, the patient's temperature reached 38.7° C and she continued to have left shoulder pain. A chest radiograph revealed left pleural effusion and computed tomography scan for ruling out subsplenic abscess was negative.

**Figure 1A and 1B:**
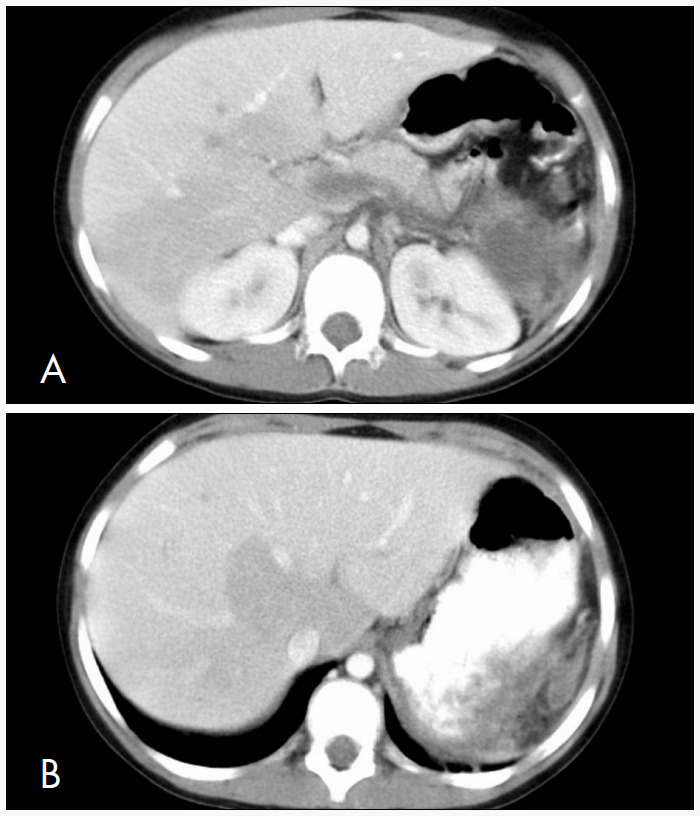
Abdominal computed tomography scan on the eighth postoperative day following laparoscopic splenectomy, showing thrombosis of the splenic vein, main portal vein, right and left portal veins, and portions of the smaller intrahepatic portal vein branches.

On the seventh postoperative day, her platelet count rose to 9,900,000, and aspirin (80 mg, four times a day) was added. On the eighth postoperative day, a repeat computed tomography scan revealed thrombosis of the splenic vein, main portal vein, right and left portal veins, and portions of the smaller intrahepatic portal vein branches (Figures 1a and 1b). Platelet-depleting pheresis was performed and anegralide (0.5 mg orally, twice a day) and enoxaparin (33 mg subcutaneously, twice a day) were administered; the aspirin dose was increased to 160 mg four times a day.

**Figure 2:**
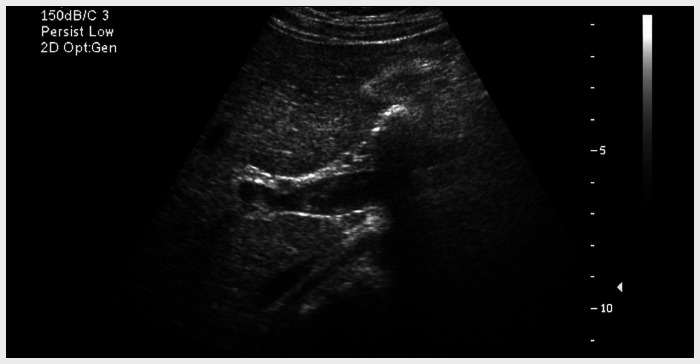
Abdominal ultrasound on eleventh postoperative day following laparoscopic splenectomy, revealing residual thrombus within the main portal vein with flow through the region, persistent thrombosis of the left portal vein, and patent right portal vein; the splenic vein was not seen.

On the eleventh postoperative day, her platelet count was 756,000. An ultrasound revealed residual thrombus in the main portal vein with flow through the region, persistent thrombosis of the left portal vein, and patent right portal vein (Figure 2). On the thirteenth postoperative day, her platelet count was 1,422,000, white blood cells 18,200, and hemoglobin 8.0. She was then discharged, to be followed up as an outpatient while on low-molecular weight heparin and hydroxyurea.

## DISCUSSION

Thrombosis of the portal or splenic vein following splenectomy is a rare event with a reported incidence of 0.2-6%.^1^ Mortality varies from 0-75%, and once the diagnosis is identified, thrombolysis and anticoagulant therapy are required. The phenomenon is more common in hematological disorders, with one retrospective series reporting 17.8% incidence of thrombosis of the portal or splenic vein after splenectomy in patients with myeloid metaplasia.^2^ Since first described in 1895 by Delatour, reports of thrombosis of these vessels have appeared in relation to patients undergoing splenectomy for myelo-proliferative disorders, hemolytic anemia, idiopathic thrombocytopenic purpura, hereditary spherocytosis and idiopathic portal vein thrombosis.^3^

The physiological basis for portal or splenic vein thrombosis is unclear, but speculation centers around hypercoagulability and stasis of the splenic/portal systems; it can occur at any time from six days to three years after the operation.^3^ While transient postoperative thrombocytosis appears in 29% of all splenectomy patients, among a group of 400 patients who underwent splenectomy as a result of trauma, none had portal vein thrombosis, which gives evidence that preexisting disease is a risk factor for this complication.^3-5^ Patients with primary myeloproliferative disorders are at increased risk for portal vein thrombosis, independent of surgical intervention, perhaps due to platelet dysfunction resulting from abnormalities of pluripotent stem cells.

Stasis in the splenic vein remnant is considered to be a mechanical risk factor and creates a large "*cul-de-sac*" of traumatized endothelium.^3-5^ As the diameter of this remnant is proportional to the size of the removed spleen, marked splenomegaly (with larger draining veins) is thought to increase the risk of thrombosis. Finally, ligation of the splenic vein is believed to decrease portal blood flow acutely, thereby resulting in thrombosis.^4^

Skarsgard et al.^5^ were the first to report splenic, portal or mesenteric venous thrombosis following splenectomy as a result of hematological disease in a pediatric population. Three patients presented with abdominal pain and nausea, with or without fever, four, eleven and thirteen days after splenectomy due to hereditary elliptocytosis, thalassemia intermedia and idiopathic thrombocytopenic purpura, respectively. These authors concluded that this diagnostic possibility should be considered for any child with abdominal pain following splenectomy and suggested that routine postoperative Doppler ultrasound performed on patients with hematological diseases treated by splenectomy should be considered, and that high-risk patients (those with massively enlarged spleens and advanced age) might benefit from aspirin prophylaxis.^5^

Broe et al.^2^ reported on cases of thrombosis of the portal and mesenteric veins following splenectomy due to myeloid metaplasia. Most patients had hypersplenism or symptomatic splenomegaly, and post-splenectomy thrombocytosis was not considered to be a risk factor. The preventative recommendations were ligation of the splenic vein close to the inferior mesenteric vein and prophylactic anticoagulation or antiplatelet therapy following the operation.^2^

Rattner et al.^3^ described portal and splenic venous thrombosis following elective splenectomy. Out of seven patients, three were initially thought to have sepsis, three pancreatitis, and one pulmonary embolus. Two patients for whom the diagnosis was not made within three days died. These authors concluded that such thrombotic events are underappreciated and should be considered as diagnostic possibilities for patients with fever and abdominal pain following splenectomy.^3^

## CONCLUSION

Clinical suspicion of portal, splenic or mesenteric vein thrombosis should be high for patients with chronic myeloid leukemia who have had splenectomy. The diagnosis is confirmed by Doppler ultrasound, contrast-enhanced computed tomography, magnetic resonance imaging magnetic resonance angiography, or arteriography. Prevention of post-splenectomy thrombosis may revolve around preoperative screening, operative technique or postoperative monitoring. During the first few days following splenectomy, the platelet levels should be monitored and, if they rise, prophylactic anticoagulation or antiplatelet therapy should be started and Doppler ultrasonography be performed. Routine ultrasonography for splenectomy patients between the sixth and twelfth postoperative days, with a second screening on the thirtieth postoperative day, is proposed. For long-term screening, it has been suggested that patients with marked splenomegaly, thrombocytosis or myeloproliferative disorder should undergo sonography examination at three, six and twelve months.^4^ The outcome after thrombosis of any of these vessels seems related to the amount of thrombosis present.
